# Evasion Mechanisms Used by Pathogens to Escape the Lectin Complement Pathway

**DOI:** 10.3389/fmicb.2017.00868

**Published:** 2017-05-12

**Authors:** Anne Rosbjerg, Ninette Genster, Katrine Pilely, Peter Garred

**Affiliations:** Laboratory of Molecular Medicine, Department of Clinical Immunology, Section 7631, Rigshospitalet, Faculty of Health and Medical Sciences, University of CopenhagenCopenhagen, Denmark

**Keywords:** innate immune system, immune evasion, complement, complement inhibition, mannose-binding lectin, ficolin, collectin, MASP

## Abstract

The complement system is a crucial defensive network that protects the host against invading pathogens. It is part of the innate immune system and can be initiated via three pathways: the lectin, classical and alternative activation pathway. Overall the network compiles a group of recognition molecules that bind specific patterns on microbial surfaces, a group of associated proteases that initiates the complement cascade, and a group of proteins that interact in proteolytic complexes or the terminal pore-forming complex. In addition, various regulatory proteins are important for controlling the level of activity. The result is a pro-inflammatory response meant to combat foreign microbes. Microbial elimination is, however, not a straight forward procedure; pathogens have adapted to their environment by evolving a collection of evasion mechanisms that circumvent the human complement system. Complement evasion strategies features different ways of exploiting human complement proteins and moreover features different pathogen-derived proteins that interfere with the normal processes. Accumulated, these mechanisms target all three complement activation pathways as well as the final common part of the cascade. This review will cover the currently known lectin pathway evasion mechanisms and give examples of pathogens that operate these to increase their chance of invasion, survival and dissemination.

## Introduction

**FIGURE 1:**
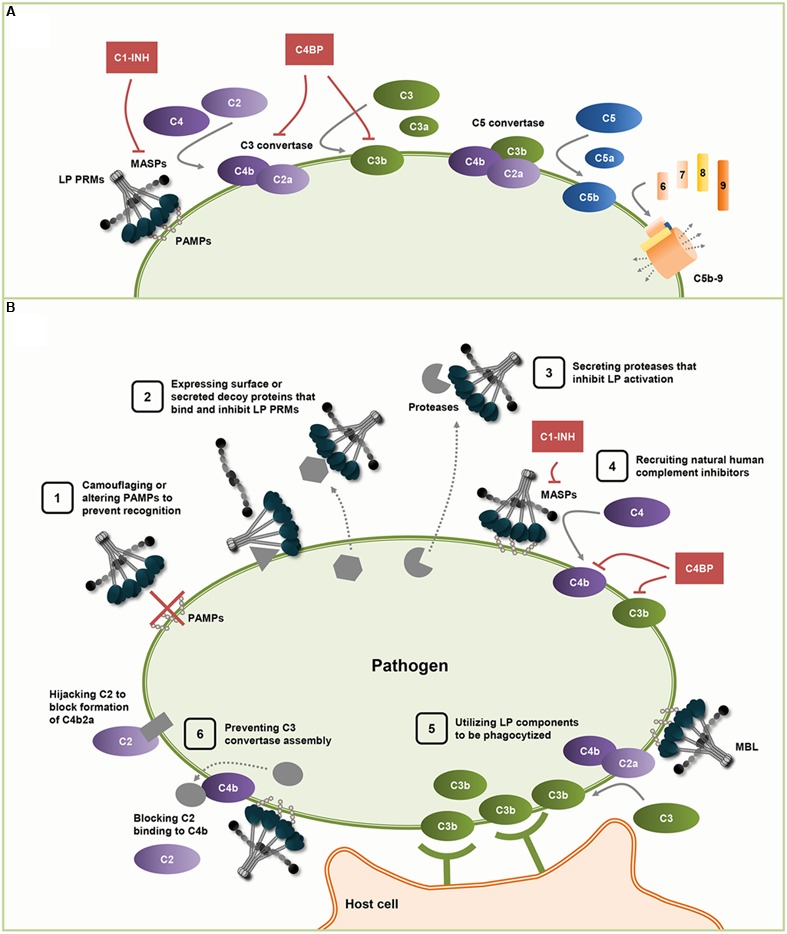
**Activation and evasion of lectin pathway (LP) of complement. (A)** The LP is activated when the pattern recognition molecules (PRMs) Mannose-Binding Lectin (MBL), ficolins or collectin-10/-11 recognize pathogen associated molecular patterns (PAMPs) on microbial surfaces. Pathogen recognition activates the PRM-associated serine proteases, MASPs, that catalyze C4 and C2 cleavage, leading to C3 convertase (C4b2a) formation. The C3 convertase cleaves C3 into the opsonin C3b and the anaphylatoxin C3a. Activation of C3 also leads to downstream formation of the C5 convertase (C4b2a3b) which cleaves C5 into the anaphylatoxin C5a and the fragment C5b. Attachment of C5b to the pathogen surface initiates formation of the lytic terminal membrane attack complex (C5b-9). The functions of these generated cleavage products include opsonization and lysis of the pathogen as well as generation of an inflammatory response. Complement inhibitory proteins like C1-INH and C4BP prevent excessive complement activation on host cells. **(B)** Microorganisms have developed multiple ways to evade complement actions and the mechanisms known to interfere with LP activation are: (1) masking of the PAMPs and thus avoiding being recognized by PRMs, (2) surface expression or secretion of proteins that bind and inhibit LP PRMs by disruption of the PRM:MASP complex and/or by impairment of PRM ligand-binding, (3) secretion of proteases that cleave and destruct LP components, (4) recruitment of the host’s complement inhibitory proteins; C1-INH that inhibits the MASP activity and C4BP that inactivates C4b, (5) utilization of LP components for voluntary opsonization by intracellular pathogens, (6) prevention of C3 convertase assembly by hijacking C2 via a surface expressed protein or by blocking the C2 binding-site on C4b via a secreted protein.

To survive within the host, successful pathogens have evolved numerous effective evasion strategies to overcome attacks from the immune system. The innate immune system, including the complement system, is the host’s first line of defense against foreign pathogens and is therefore crucial in determining outcome of a pathogen-host confrontation. The complement system consists of a network of plasma proteins that trigger a proteolytic cascade upon activation (Ricklin et al., 2010). Three pathways initiate complement activation: the lectin (LP), classical (CP) and alternative (AP) pathways. The LP is initiated when the pattern recognition molecules (PRMs) Mannose-Binding Lectin (MBL), ficolins (ficolin-1, -2 and -3) or collectin-10/-11 recognize carbohydrate ligands that are specifically present on microbial surfaces (**Figure 1A**) (Garred et al., 2016). The LP PRMs are soluble multimeric molecules consisting of a collagen-like domain and a carbohydrate-binding domain. Similar to C-type lectin receptors expressed on the myeloid cell surface, the ligand specificities of LP PRMs include carbohydrates such as mannose, *N*-acetylglucosamine and β-glucan (Dambuza and Brown, 2015; Garred et al., 2016). The PRMs form a complex with associated serine proteases named MASP-1, -2 and -3 that are activated upon pathogen recognition. These complexes catalyze C4 and C2 cleavage, leading to C3 convertase (C4b2a) formation. The C3 convertase cleaves C3 into the opsonin C3b and the anaphylatoxin C3a. Activation of C3 also leads to downstream formation of the C5 convertase (C4b2a3b) which cleaves C5 into the anaphylatoxin C5a and the fragment C5b; the latter attach to the pathogen surface and initiates the terminal membrane attack complex (C5b-9). The CP is activated when the complement protein C1q recognizes antigen-antibody complexes on foreign surfaces and its associated serine proteases cleave C4 and C2 to generate the C3 convertase. The AP is activated by spontaneous hydrolysis of C3 and through a C3-driven amplification loop leading to formation of the alternative C3 convertase. Once the C3 convertase is formed, all subsequent steps are common for the three activation pathways. Regardless of activation mode, the cascade of events leads to elimination of the intruder by formation of cleavage products that function in opsonization and lysis of the pathogen as well as generation of an inflammatory response (Ricklin et al., 2010).

As a countermove, several pathogens possess efficient mechanisms to avoid these defensive strategies from the host, and many diverse pathogens share common general mechanisms to avoid complement attack (Lambris et al., 2008; Zipfel et al., 2013; Garcia et al., 2016). These evasion mechanisms can counteract different events in the entire complement system, but this review will focus on the mechanisms that interfere with activation of the LP, i.e., mechanisms that interact with steps leading to formation of the C3 convertase by LP (**Figure 1B**). The following sections will provide an overview of mechanisms that involve; avoiding LP recognition molecules, exploiting LP components and preventing C3 convertase assembly.

## Avoiding Lp Recognition Molecules

### Masking of PAMPs

The surface structure of a pathogen provides a signature for recognition by the host, e.g., MBL and ficolins recognize pathogen associated molecular patterns (PAMPs) on the microbial surface, which lead to LP complement activation. Accordingly, one evasion strategy employed by pathogens is to camouflage or alter the surface of the microbe (or the infected cell) in order to hide from the host surveillance systems (**Figure 1B** (1)). This strategy is used by certain *Klebsiella pneumoniae* strains that can alter their capsular composition to prevent recognition by the LP (Sahly et al., 2009). It was shown that Klebsiella-induced respiratory burst in phagocytes occurs via AP and LP. However, Klebsiella serotypes that lack expression of capsular polysaccharides containing mannobiose or rhamnobiose, which are recognized by LP PRMs, induce lower respiratory burst in phagocytes than those expressing the glycoepitopes. Additionally, these serotypes are more likely to evade intracellular killing by phagocytes. Therefore, lack of these glycoepitopes benefits the pathogen.

### Surface Expression of Decoy Proteins

Another strategy utilized by pathogens to avoid LP complement activation is to express a protein on its surface that binds directly to and inhibit the LP recognition molecules (**Figure 1B** (2)). One example of this type of LP inhibitor is found in human astroviruses (HAstVs); a coat protein composing the viral capsid of the virus binds to MBL and thereby inhibits activation of LP on mannan (Hair et al., 2010). HAstV Coat Protein binds to wildtype MBL, but not to a variant of MBL mutated in a lysine residue (Lys55) critical for binding to MASP-2. Hence, it appears that Coat Protein blocks the serine protease binding region of MBL, which interrupts the normal association of MBL with MASP-2.

A somewhat similar LP inhibitory mechanism is employed by the intracellular parasite *Trypanosoma cruzi*, the causal agent of Chagas’ disease (Ferreira et al., 2004; Sosoniuk et al., 2014). First it was reported that *T. cruzi* calreticulin (TcCRT), a chaperone molecule that translocates from the ER to the parasite surface, binds specifically to the collagen-like domain of MBL resulting in impaired MBL-binding to its ligand mannose. However, the impaired ligand binding of MBL caused by TcCRT did not induce any functional consequence in LP activation (Ferreira et al., 2004). Later it was reported that TcCRT also binds to the collagen-like domain of another LP PRM, ficolin-2, resulting in inhibition of ficolin-2 mediated LP activation, although the interaction did not impair the ligand-binding capacity of ficolin-2 (Sosoniuk et al., 2014). Whether a direct TcCRT-MASP interaction takes place, with or without release of the MASPs, was not investigated. Nevertheless, these data indicate that *T. cruzi* uses TcCRT to inhibit LP activation as a survival strategy.

### Secretion of Decoy Proteins

Pathogens can also utilize secreted proteins to inhibit LP activation (**Figure 1B** (2)). One example of a virus-encoded inhibitor is the Flavivirus non-structural protein 1 (NS1); a glycoprotein secreted from dengue virus (DENV) that binds MBL (Thiemmeca et al., 2016). In the absence of NS1 MBL binds directly to DENV and prevents attachment and entry of virus to host cells. However, soluble NS1 is released from DENV-infected cells together with DENV virions and the competitive binding of NS1 to MBL protects the virus from MBL-mediated neutralization, independent of complement activation.

Other secreted pathogen-derived LP PRM inhibitory proteins include the scabies mite inactivated protease paralogs (SMIPPs). Scabies mites feed on epidermal protein and the SMIPPs are a family of catalytically inactive serine proteases secreted by the mites. The SMIPPs inhibit all three complement pathways and their inhibitory action is due to binding of C1q, properdin and MBL (Bergström et al., 2009). Specifically two SMIPPs, D1 and I1, bind directly to MBL (but not to MASPs) and inhibit downstream activation (Reynolds et al., 2014), but their mechanism of action is different; binding of D1 to the MBL:MASP complex releases MASP-2 from the complex, whereas binding of I1 does not. Regardless of the mechanism, both molecules seem to provide a favorable situation for the mite, in terms of avoiding LP activation.

An alternative strategy to avoid LP recognition is when a vector-borne pathogen co-opts a vector protein with inhibitory action on LP. *Borrelia burgdorferi*, the Lyme disease agent, is transmitted to vertebrates by ticks during the blood meal. In order to suppress the immune system of the host, ticks secrete salivary proteins at the bite-site. Among these proteins is the Tick Salivary Lectin Pathway Inhibitor (TSLPI) (Schuijt et al., 2011). As implied by its name, TSLPI impairs complement-dependent killing through specific inhibition of the LP, which benefits *B. burgdorferi*. Although CP and AP have shown to be important for *B. burgdorferi* elimination, neither is inhibited by TSLPI. The inhibitory effect on LP appears to be by prevention of MBL ligand binding rather than impairment of MASP-2 activity. Deglycosylation of TSLPI decreases the inhibitory effect of the protein, suggesting that it binds to the carbohydrate recognition domains of MBL. In addition, TSLPI reduced ficolin-2 ligand binding, thereby inhibiting complement activation.

### Enzymatic Cleavage of PRMs

Pathogen-derived proteases degrade complement components into smaller non-functional fragments (**Figure 1B** (3)). Evidence of such proteases that cleave LP PRMs comes from *Tannerella forsythia*, the main cause of periodontitis. *T. forsythia*, produces two metalloproteinases named Karilysin and Mirolysin that degrade MBL, ficolin-2, ficolin-3 and C4, thereby inhibiting LP (and CP) (Jusko et al., 2012, 2015). *T. forsythia* mutants lacking the two proteases show reduced survival in serum, indicating that complement inactivation is a crucial survival strategy for this pathogen (Jusko et al., 2015).

## Exploiting Lp Components

### Recruiting Natural Human Complement Inhibitors

Some microorganisms have evolved an ability to recruit natural human complement inhibitors to their surface and thus mimic the way host cells prevent excessive complement activation (**Figure 1B** (4)). The known human inhibitors affecting the LP are C1-inhibitor (C1-INH), C4b-binding protein (C4BP), MBL/ficolin/CL-associated protein-1 (MAP-1) and small MBL-associated protein (sMAP) (Schmidt et al., 2016). Besides, a high degree of overlap between complement and coagulation means that coagulation inhibitors can affect LP, e.g., anti-thrombin inactivation of MASP-1 and -2 (Presanis et al., 2004).

*Escherichia coli* and *Bordetella pertussis* are examples of pathogens that recruit and utilize C1-INH to evade complement (Lathem et al., 2004; Marr et al., 2007). C1-INH was discovered as an inhibitor of the C1 complex (C1qr_2_s_2_: C1q and its associated proteases), but it also targets LP complexes consisting of PRMs and MASPs. Thus, if a pathogen manipulates C1-INH it will probably disturb both pathways if these are active.

C4b-binding protein works as a cofactor in cleavage and inactivation of C4b and C3b and many pathogens exploit C4BP as part of their survival strategy, which has been thoroughly described in previous reviews (Blom and Ram, 2008; Hovingh et al., 2016). *Leptospira interrogans* binds C4BP via its surface molecule Lsa23 and induce C4b and C3b degradation (Siqueira et al., 2016) (**Figure 1B** (4)). Interestingly, Lsa23 is also able to attract plasminogen, which after activation into plasmin was shown to directly cleave C4b and C3b (Siqueira et al., 2016). This demonstrates that cross-talk between complement and coagulation also exists in immune evasion.

### Utilizing LP Components to be Phagocytized

*Leishmania* is a family of parasites transferred to humans via sand flies causing visceral and cutaneous leishmaniasis. *Leishmania* parasites can survive inside human macrophages and it has therefore been of interest to identify molecules involved in the interaction between the two. MBL was suggested as a candidate because (i) MBL binds to *Leishmania* (Green et al., 1994) (ii) it has been speculated whether some degree of positive selection for low MBL individuals exist since a high frequency of variant alleles causing lowered MBL levels are sustained in many populations – the hypothesis being that MBL mediates phagocytosis of pathogens able to reside inside phagocytes (Garred et al., 1994). Case-control studies of visceral leishmaniasis have concluded that the risk of infection is decreased in individuals with genotypes associated with low MBL levels (Alonso et al., 2007; Mishra et al., 2015), whereas a study of cutaneous leishmaniasis showed the opposite (Araujo et al., 2015). Increased MBL-driven macrophage uptake was not confirmed in the visceral leishmaniasis studies; hence, it is not clear if MBL acts as a direct opsonizer or if it mediates downstream C3b deposition (**Figure 1B** (5)). A third hypothesis has also been proposed: *in vitro* experiments have shown that ingestion of MBL-opsonized *L. chagasi* stimulates macrophages to secrete more TNF-α and IL-6 than non-opsonized parasites. This MBL-mediated secretion was hypothesized to guide the subsequent T-cell development in a parasite-favorable direction (Santos et al., 2001). On the contrary, a study of *Blastomyces dermatitidis* showed that MBL opsonization downregulated the TNF-α secretion by macrophages and in this case a downregulation of TNF-α was regarded as an advantage for the pathogen (Koneti et al., 2008). Hence, consequences from a pathogen attack/immune response can be difficult to interpret as the same immune response has different effects depending of the pathogen.

*Mycobacterium tuberculosis* also binds MBL (Bartlomiejczyk et al., 2014) and has developed a strategy of hiding inside macrophages by preventing lysosomal degradation (Flynn and Chan, 2003). Case-control studies of tuberculosis infection have, however, pointed in different directions; some show that MBL increases susceptibility (Hoal-Van Helden et al., 1999; Søborg et al., 2003; Selvaraj et al., 2006) and others show that MBL is protective (Capparelli et al., 2009; Chen et al., 2015; Liu et al., 2016) or insignificant (Chalmers et al., 2015). The reason for the discrepancy could perhaps be found in the differences of assessing MBL genotypes and timing of the blood sampling for measuring MBL serum levels. Hence, the role of MBL in tuberculosis remains an open question.

Also human immunodeficiency virus (HIV) has been speculated to use voluntary opsonization. Like all viruses HIV utilizes the transcriptional machinery of the host cell to amplify its genetic material. Complement activation on HIV mediates deposition of C3b, which leads to phagocytosis (Thieblemont et al., 1993; Bajtay et al., 2004) and because of MBL’s ability to bind and activate complement on HIV, MBL may enhance infection (Haurum et al., 1993; Saifuddin et al., 2000). A case-control study have shown that low MBL levels are associated with delayed AIDS onset (Maas et al., 1998) and the same has been shown for MASP-2 (Boldt et al., 2016). Paradoxical, the latter study also showed that the risk of getting the initial HIV infection was increased with low MASP-2 levels (Boldt et al., 2016). The stage of disease probably determines whether these LP molecules represent an advantage or disadvantage for the host (Prohászka et al., 1997) and perhaps explains why other studies have found MBL to be a protective factor against HIV (Garred et al., 1997; McBride et al., 1998).

## Prevent C3 Convertase Assembly

### Hijacking of C2

The parasites *Schistosoma* and *Trypanosoma* express a surface protein that enables them to avoid complement attack by LP and CP (Inal and Sim, 2000; Cestari et al., 2008). The molecule was first described under the name sh-TOR (Inal, 1999), but is now known as Complement Receptor Inhibitor Trispanning (CRIT). CRIT binds C2 via its extracellular domain and thereby hinder C2 binding to C4b, thus compromising C3 convertase (C4b2a) formation (**Figure 1B** (6)). CRIT is an example of molecular mimicry as it has been reported that CRIT binds C2 with a domain homologs to a region on human C4b (Inal and Schifferli, 2002). Both LP and CP are disrupted when C2 is hijacked, but *T. cruzi* specifically evades LP since complement on *T. cruzi* is shown to predominantly be activated via this pathway (Evans-Osses et al., 2013).

### Blocking of C4b

The bacteria *Staphylococcus aureus* causes severe diseases like toxic shock syndrome and includes methicillin-resistant *S. aureus* (MRSA) strains. *S. aureus* has a palette of evasion mechanisms and possibly one is to reduce the LP and CP activity using a protein called extracellular adherence protein (Eap). Eap binds C4b and blocks assembly of the C3 convertase C4b2a (Woehl et al., 2015) (**Figure 1B** (6)). After secretion, a fraction of Eap rebinds *S. aureus*, but it is the fluid phase Eap that forms complexes with C4b. In fact, experiments showed that only exogenously added Eap reduced opsonization/phagocytosis and *S. aureus* were not more susceptible to phagocytosis after knocking out endogenous Eap (Woehl et al., 2015). This questions whether the purpose of Eap is to inhibit LP and CP. It has been shown that patients with *S. aureus* infections have high titers of anti-Eap antibodies confirming the importance of the protein (Joost et al., 2011), but Eap is a multifaceted protein with many functions in *S. aureus* virulence, which can explain the reported antibody titers (Harraghy et al., 2003).

A functional equivalent to Eap named complement interfering protein (CIP) is secreted by *Streptococcus agalactiae*, which is a bacterium that can be transmitted from mother to child during pregnancy and cause severe neonatal disease. The amino acid sequence of CIP is 15% identical to Eap and the function of CIP is also to bind C4b and obstruct C3 convertase formation (Pietrocola et al., 2016).

## Concluding Remarks

Evasion mechanisms are found to interfere with different steps of the LP cascade, from PRMs to C3 convertase formation. It is a complex field as immune evasion and protection by the host immune system sometimes represent two sides of the same coin, e.g., MBL mediated opsonization. Evolution has equipped microorganisms and humans with neutralizing and utilizing countermoves against one another, but some microorganisms are one step ahead, which makes them pathogenic. These are the mechanisms important to investigate and probably more ways of evading LP will be discovered in the near future. Studies on mechanisms of immune evasion and complement inhibition will provide pivotal insight into host-pathogens confrontations and hopefully lead to better treatment for various human infectious diseases.

## Author Contributions

AR and NG wrote the paper. KP prepared the figures. PG performed a critical revision. All authors prepared the outline of the article, read and approved the final manuscript.

## Conflict of Interest Statement

The authors declare that the research was conducted in the absence of any commercial or financial relationships that could be construed as a potential conflict of interest.
